# Diagnostic yield of TOF-MRA for detecting incidental vascular lesions in patients with cognitive impairment: An observational cohort study

**DOI:** 10.3389/fneur.2022.958037

**Published:** 2022-08-25

**Authors:** Ho Young Park, Chong Hyun Suh, Woo Hyun Shim, Hwon Heo, Woo Seok Kim, Jae-Sung Lim, Jae-Hong Lee, Ho Sung Kim, Sang Joon Kim

**Affiliations:** ^1^Department of Radiology and Research, Asan Medical Center, Institute of Radiology, University of Ulsan College of Medicine, Seoul, South Korea; ^2^Department of Neurology, Asan Medical Center, University of Ulsan College of Medicine, Seoul, South Korea

**Keywords:** cognitive dysfuction, magnetic resonance angiography (MRA), intracranial artery stenosis, extracranial artery stenosis, cerebral aneurysm, observational study

## Abstract

**Objectives:**

The role of three-dimensional (3D) TOF-MRA in patients with cognitive impairment is not well established. We evaluated the diagnostic yield of 3D TOF-MRA for detecting incidental extra- or intracranial artery stenosis and intracranial aneurysm in this patient group.

**Methods:**

This retrospective study included patients with cognitive impairment undergoing our brain MRI protocol from January 2013 to February 2020. The diagnostic yield of TOF-MRA for detecting incidental vascular lesions was calculated. Patients with positive TOF-MRA results were reviewed to find whether additional treatment was performed. Logistic regression analysis was conducted to identify the clinical risk factors for positive TOF-MRA findings.

**Results:**

In total, 1,753 patients (mean age, 70.2 ± 10.6 years; 1,044 women) were included; 199 intracranial aneurysms were detected among 162 patients (9.2%, 162/1,753). A 3D TOF-MRA revealed significant artery stenoses (>50% stenosis) in 162 patients (9.2%, 162/1,753). The overall diagnostic yield of TOF-MRA was 16.8% (294/1,753). Among them, 92 patients (31.3%, 92/294) underwent either medical therapy, endovascular intervention, or surgery. In total, eighty-one patients with stenosis were prescribed with either antiplatelet medications or lipid-lowering agent. In total, fifteen patients (aneurysm: 11 patients, stenosis: 4 patients) were further treated with endovascular intervention or surgery. Thus, the “number needed to scan” was 19 for identifying one patient requiring treatment. Multivariate logistic regression analysis showed that being female (odds ratio [OR] 2.05) and old age (OR 1.04) were the independent risk factors for intracranial aneurysm; being male (OR 1.52), old age (OR 1.06), hypertension (OR 1.78), and ischemic heart disease history (OR 2.65) were the independent risk factors for significant artery stenosis.

**Conclusions:**

Our study demonstrated the potential benefit of 3D TOF-MRA, given that it showed high diagnostic yield for detecting vascular lesions in patients with cognitive impairment and the considerable number of these lesions required further treatment. A 3D TOF-MRA may be included in the routine MR protocol for the work-up of this patient population, especially in older patients and patients with vascular risk factors.

## Introduction

Brain magnetic resonance imaging (MRI) plays a crucial role in the diagnostic work-up and follow-up of patients with cognitive impairment (1, 2). With the wider use of brain MRI in clinical practice, the cases of incidental imaging findings in patients with cognitive impairment have been increasing (3, 4). Studies have indicated that positive findings have been more frequently observed in patients with a cognitive impairment compared to healthy elderly, although majority of these incidental findings do not indicate the requirement of any treatment (3). However, incidental vascular findings such as aneurysm or stenosis are not well documented in the literature. Detection of extra- or intracranial artery stenosis and intracranial aneurysm in patients with cognitive impairment might be important because these disorders may lead to stroke or subarachnoid hemorrhage, imposing significant burden on patients and their families.

TOF (time of flight)-MRA is the most commonly used MRA technique (5). One of the main advantages of this sequence is that it does not require a contrast agent for imaging. Thus, it can be readily added to brain MR protocols for vascular survey. It is also suitable for MR protocols for patients with cognitive impairment because the current optimal protocols do not recommend contrast administration unless other suspicious imaging findings are present (6). Nevertheless, the optimal protocols do not include TOF-MRA as a routine sequence (6, 7). Several studies have demonstrated the association between cognitive impairment and atherosclerosis (8–10). Not only vascular dementia but also Alzheimer's disease is significantly associated with the atherosclerosis of neck vessels or intracranial arteries; however, the underlying pathophysiology of this association remains poorly understood (11). Moreover, a few studies have reported the possibility of an association between cognitive impairment and intracranial aneurysms, given the fact that a non-negligible proportion of patients have cognitive impairment even before undergoing treatment for aneurysm (12, 13). However, vascular lesions in these patients might be overlooked without TOF-MRA.

Risk factors for intracranial aneurysm and vascular stenosis in general population are well established in the previous studies. Female sex, hypertension, and smoking are the well-known risk factors for intracranial aneurysm (14–16). In addition, male sex, hypertension, diabetes mellitus, and dyslipidemia are the significant risk factors for intracranial artery stenosis (17–19). However, risk factor analysis for the vascular lesions in cognitive impairment patients is currently absent, and there might be a difference in risk factors in this patient group. Risk factor stratification is important in patients with cognitive impairment to determine which patient group should undergo TOF-MRA or not.

Therefore, we aimed to explore the clinical utility of TOF-MRA in patients with cognitive impairment by evaluating its diagnostic yield for detecting incidental extra- or intracranial artery stenosis and intracranial aneurysm. Furthermore, we aimed to identify the clinical risk factors for positive TOF-MRA findings in this patient population.

## Methods

This retrospective, observational, single-institution study was approved by the institutional review board of Asan Medical Center, and the need for informed consent was waived. We reported our results according to the guidelines of Strengthening the Reporting of Observational Studies in Epidemiology (STROBE) (20).

### Patient inclusion

From January 2013 to February 2020, patients complaining cognitive impairment who underwent a dedicated brain MRI protocol for dementia at our institution were consecutively enrolled through a retrospective review of our electronic database. Whether to perform TOF-MRA or not was determined according to the referring physicians' preference considering patients' medical history such as hypertension, diabetes mellitus, or coronary artery disease. Patients were excluded if (1) they did not undergo TOF-MRA; (2) their images were of poor quality; and (3) they already knew the existence of aneurysms or stenoses.

### Imaging protocol

Magnetic resonance imaging was performed using a 3.0-T system (Ingenia; Philips Medical Systems, Best, The Netherlands) with an eight-channel head coil. The dementia protocol at our institution included the following sequences: a three-dimensional (3D) T1-weighted image, two-dimensional T2-weighted image, two-dimensional fluid-attenuated inversion recovery image, susceptibility-weighted image, diffusion tensor image, and separate 3D-TOF-MRA image for carotid and intracranial vessel. The scan time for TOF-MRA for the head and the carotids was approximately 4 min and 30 s in total. Gadolinium-enhanced T1-weighted imaging was performed if necessary. The detailed parameters are presented in the Supplementary Table 1.

### MRI analysis

Brain MRI with TOF-MRA was reviewed by two authors (H.Y.P. and C.H.S. with 6 and 11 years of experience in diagnostic radiology, respectively). Only saccular aneurysms were counted and their number, size, and location were documented. Aneurysms were categorized based on the size into the groups of <3, 3–5, 5–7, 7–10, and >10 mm according to the previous studies based on the risk stratification for spontaneous rupture (21, 22). Degree of stenosis was calculated and significant stenosis was defined as >50% stenosis on MRA. Degree of stenosis was divided into moderate stenosis (50–69%), severe stenosis (70–99%), and total occlusion according to a previous study (23). The locations of stenosis were categorized as follows: extracranial ICA or carotid bulb, intracranial ICA, ACA, MCA, PCA, VA, and BA. If multiple stenoses were identified, the most severe stenosis was reported. The detailed methods of evaluation are summarized in the Supplementary materials. In total, two radiologists (H.Y.P. and C.H.S.) reached a consensus in case of ambiguous results.

### Outcome

The primary outcome was the diagnostic yield of TOF-MRA for detecting incidental vascular lesions in patients with cognitive impairment. Diagnostic yield was defined as the proportion of patients with positive findings of incidentally detected intracranial aneurysm or extra- and intracranial artery stenoses among those who underwent brain MRI with TOF-MRA for cognitive impairment work-up. Patients with positive TOF-MRA findings were reviewed to identify whether additional treatment was performed and the “number needed to scan (NNS)” was calculated. The number needed to scan is a similar concept to “number needed to treat,” and it indicates the number of TOF-MRA examinations needed to be performed to identify one patient who requires subsequent vascular intervention. The secondary outcomes were the clinical risk factor identification and risk stratification for positive TOF-MRA findings in patients with cognitive impairment.

### Statistical analysis

The diagnostic yield of TOF-MRA was calculated as the number of patients with positive MRA findings divided by the total number of enrolled patients. In addition, the diagnostic yield of TOF-MRA was calculated in a subset of the patients with Clinical Dementia Rating (CDR) ≥ 0.5. The cognitive scales [Mini-Mental State Examination (MMSE) and Global Deterioration Scale (GDS)] were compared between the patients with and without vascular lesions on TOF-MRA. Logistic regression analysis was performed to identify the risk factors for positive MRA findings. Sex, age, and vascular risk factors including hypertension, dyslipidemia, diabetes mellitus, smoking, alcohol, obesity, and stroke or ischemic heart disease history were used as variables in the univariate analysis. Variables with *p* < 0.1 in the univariate analysis were then entered into multivariate analysis using backward elimination method based on the maximum partial likelihood estimation. At each step, variables with *p* > 0.1 were removed. Vascular risk factors were chosen based on the previous large cohort studies (17, 18). Hypertension, dyslipidemia, diabetes, and atrial fibrillation were defined according to the documentation of these diagnoses on the patients' electronic medical chart or drug intake for these disorders. Smoking habit was considered present if a patient was a current smoker or if time interval since abstinence was <5 years. Obesity was defined as a body mass index > 30 kg/m^2^. Alcohol consumption was considered positive based on the patients' response from the medical charts. Since referring physicians' preference was involved in performing TOF-MRA, there was a potential risk of selection bias. To evaluate the presence of selection bias, we compared the characteristics of patients between the groups that underwent and did not undergo TOF-MRA. *p*-values were adjusted for multiple comparisons using the Benjamini–Hochberg method, and the false discovery rate-adjusted *p*-values were obtained (24). The adjusted *p*-values < 0.05 were considered to be statistically significant. All statistical analyses were performed using SPSS version 23 (SPSS; Chicago, IL, USA) and R Statistical Software version 4.0.5.

## Results

### Patient's characteristics

**Figure 1:**
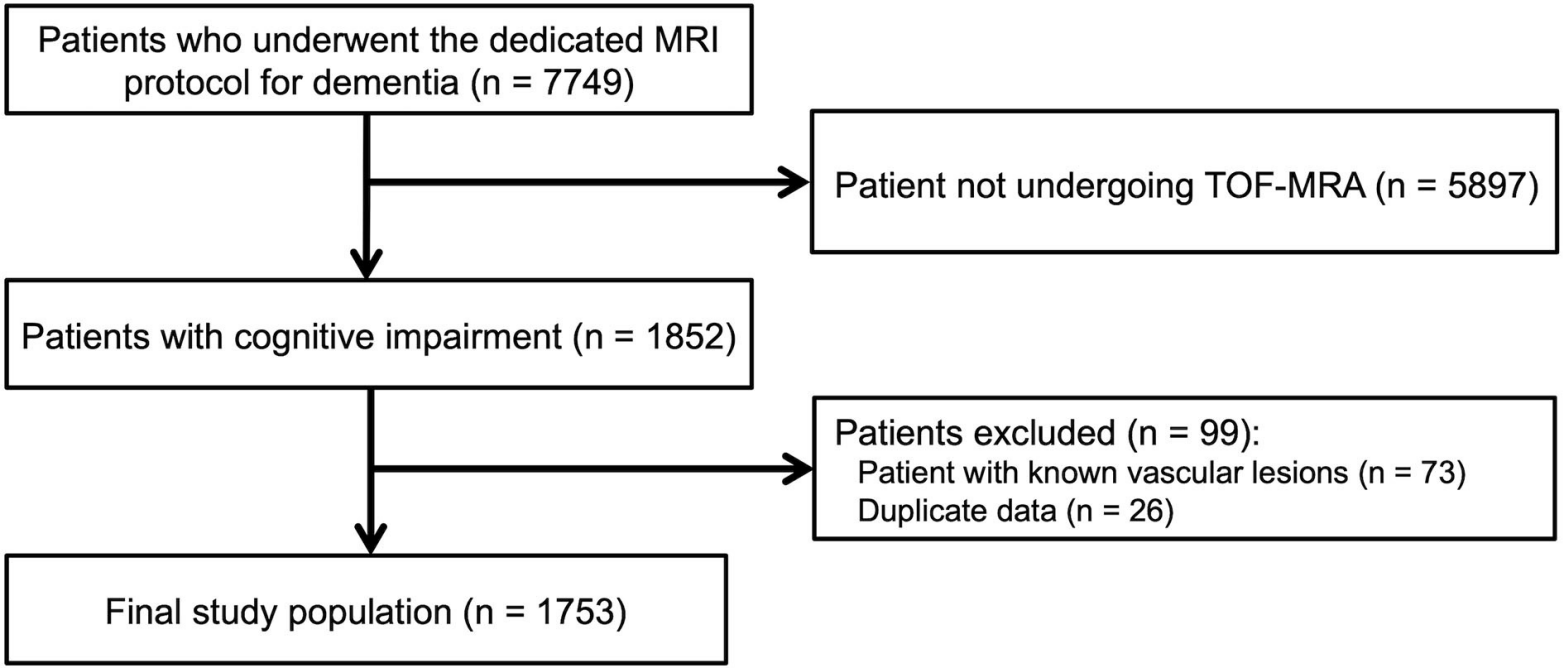
Flow diagram of patient inclusion. TOF-MRA, time-of-flight magnetic resonance angiography.

A total of 7,749 patients underwent our dedicated MRI protocol for dementia between January 2013 and February 2020. Among them, 5,897 patients were excluded because they did not undergo TOF-MRA. Furthermore, 73 patients with known vascular lesions before MRI and 26 patients of duplicate data were excluded from the analysis. Finally, 1,753 consecutive patients (mean age, 70.2 ± 10.6 years; 1,044 women) were included (Figure 1). The demographics of the included patients are presented in Table 1. The characteristics between the included and excluded patients are summarized in the Supplementary Table 2. Briefly, the included group showed higher proportion of the patients with positive vascular risk factors including hypertension, diabetes, dyslipidemia, and obesity. However, the absolute differences between the groups were not large, ranging from 1.6 to 8.3%.

**Table 1:** Patient demographics.

**Characteristics**	**Patients with cognitive impairment** **(*n* = 1,753)**
Sex (*n*)	
Female	1,044 (59.6%)
Male	709 (40.4%)
Age (years)	70.2 ± 10.6 (mean standard ± deviation)
Education (years)	9.7 ± 5.4
MMSE (0–30 points)	23.9 ± 5.5
GDS (1–7 scales)	3.2 ± 2.1
CDR (0–3 scales)	0.6 ± 0.5
Vascular risk	
Hypertension	980 (55.9%)
Diabetes	513 (29.3%)
Dyslipidemia	567 (32.3%)
Smoking	419 (23.9%)
Alcohol	555 (31.7%)
Obesity	59 (3.4%)
Previous stroke	110 (6.3%)
Ischemic heart disease	127 (7.2%)

### TOF-MRA features of intracranial aneurysms

The TOF-MRA features of 199 aneurysms from 162 patients are summarized in Table 2. Patients with a single aneurysm were most common (79.6%, 129/162), followed by those with double (17.9%, 29/162) and triple (2.5%, 4/162) aneurysms. Most aneurysms measured <7 mm (97.0%, 193/199). A number of five aneurysms measured 7–10 mm, and one aneurysm measured >10 mm. Intracranial aneurysms were most frequently located in ICA and MCA (68.3%, 136/199), followed by posterior and anterior communicating arteries (18.6%, 37/199), BA and VA (7.5%, 15/199), ACA (3.5%, 7/199), and PCA (2.0%, 4/199).

**Table 2:** TOF-MRA features of intracranial aneurysms.

**Feature**	**Value**
Female	117 (72.2%)
Male	45 (27.8%)
**Number of aneurysm(s) in a patient**	
Single	129 (79.6%)
Double	29 (17.9%)
Triple	4 (2.5%)
**Size**
<3 mm	99 (49.7%)
3–5 mm	75 (37.7%)
5–7 mm	19 (9.5%)
7–10 mm	5 (2.5%)
≥10 mm	1 (0.5%)
**Location**	
ICA	103 (51.8%)
MCA	33 (16.6%)
Pcom	25 (12.6%)
Acom	12 (6.0%)
BA	8 (4.0%)
ACA	7 (3.5%)
VA	7 (3.5%)
PCA	4 (2.0%)

### TOF-MRA features of extra- and intracranial artery stenoses

The TOF-MRA features of significant extra- and intracranial artery stenoses in 162 patients are presented in Table 3. Patients with severe stenosis were most common (44.4%, 72/162), followed by those with moderate stenosis (30.2%, 49/162) and total occlusion (25.3%, 41/162). The locations of stenoses were intracranial in 107 patients (66.0%, 107/162) and extracranial in 55 patients, all located in the carotid bulb (34.0%, 55/162). Among the intracranial stenoses, MCA and VA were the most common location (16.7% for each location, 27/162), followed by ICA (13.6%, 22/162), and PCA (11.7%, 19/162). Significant stenoses in BA and ACA were observed in only a small proportion of the study population (7.4%, 12/162).

**Table 3:** TOF-MRA features of significant extra- and intracranial artery stenosis.

**Feature**	**Value**
Female	78 (48.1)
Male	84 (51.9)
**Degree of stenosis**	
Moderate stenosis	49 (30.2)
Severe stenosis	72 (44.4)
Occlusion	41 (25.3)
**Location**	
Extracranial ICA or carotid bulb	55 (34.0)
Intracranial ICA	22 (13.6)
MCA	27 (16.7)
VA	27 (16.7)
PCA	19 (11.7)
ACA	7 (4.3)
BA	5 (3.1)

### Diagnostic yield of TOF-MRA

Among the 1,753 patients with cognitive impairment, TOF-MRA revealed incidental intracranial aneurysms in 162 patients (117 women, 45 men) and significant extra- or intracranial artery stenoses in 162 patients (78 women, 84 men). A total of thirty patients had both aneurysms and significant stenoses. The overall diagnostic yield of TOF-MRA for detecting incidental vascular lesions in patients with cognitive impairment was 16.8% (294/1,753; 95% CI 15.1–18.6%); furthermore, the diagnostic yields for detecting incidental intracranial aneurysms and extra- and intracranial artery stenoses were both 9.2% (162/1,753; 95% CI 7.9–10.7%). In patients with CDR ≥ 0.5, similar results were obtained with the overall diagnostic yield of 18.3% (134/734; 95% CI 15.5–21.2%); the diagnostic yields for intracranial aneurysms and stenoses are 9.0% (66/734; 95% CI 7.0–11.3%) and 11.6% (85/734; 95% CI 9.4–14.1%), respectively. Patients with vascular lesions showed worse cognitive function than patients without vascular lesions (MMSE 24.2 vs. 22.5, *p* < 0.001). In addition, the proportion of moderate dementia (GDS ≥ 4) was significantly higher in the patients with vascular lesions (42.1 vs. 26.7%, *p* < 0.001).

**Figure 2:**
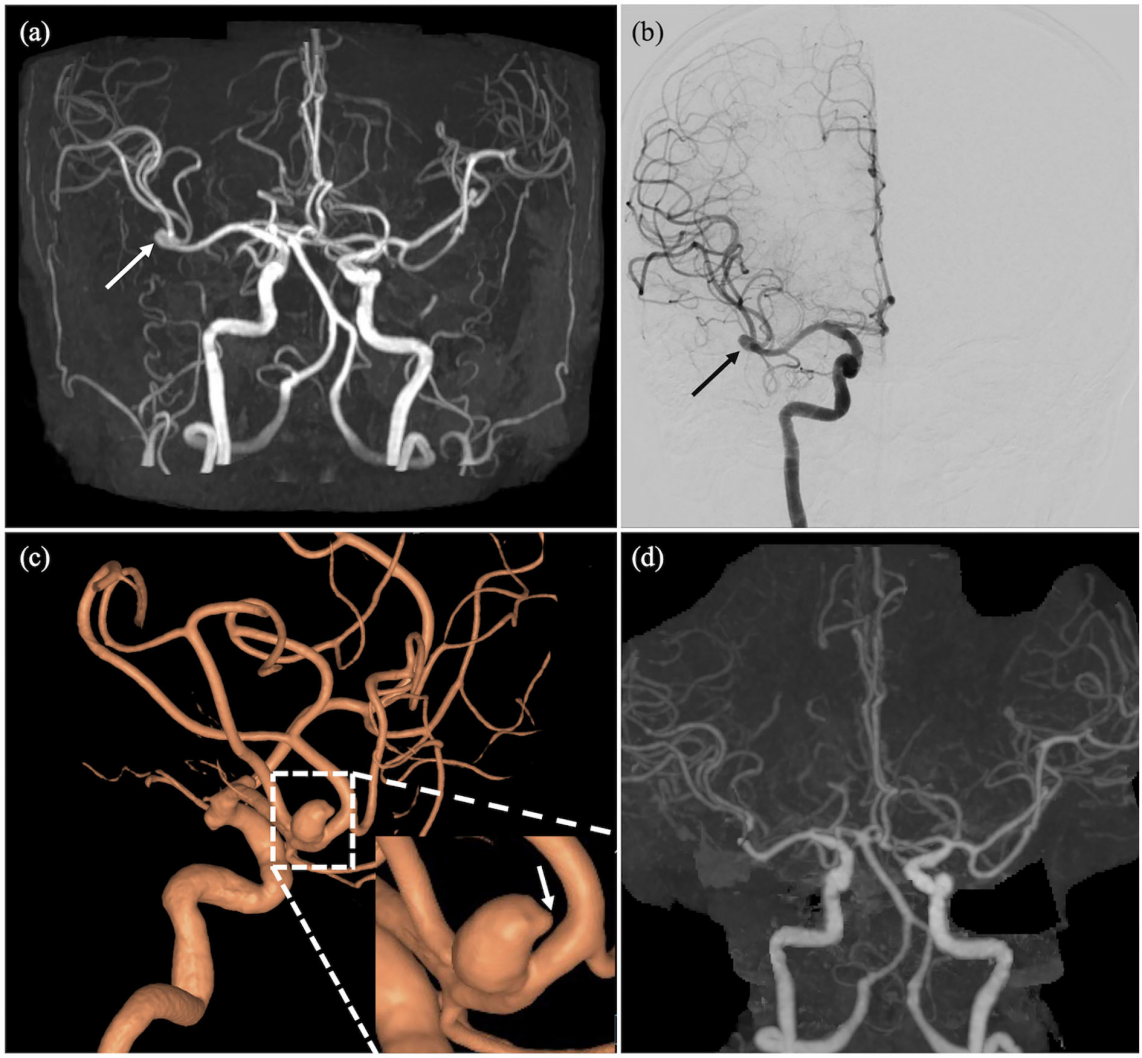
An incidentally detected intracranial aneurysm in a 75 year-old-female with cognitive impairment. A 6.5-mm aneurysm (arrows) at right MCA bifurcation on TOF-MRA **(a)** and digital subtraction angiography **(b)**. A 3D reconstruction of a digital subtraction angiography of right ICA demonstrating the aneurysm at right MCA bifurcation with a bleb formation (arrow) at the aneurysmal dome **(c)**. TOF-MRA after surgical clipping shows no filling within the aneurysm and patent distal flow **(d)**.

**Figure 3:**
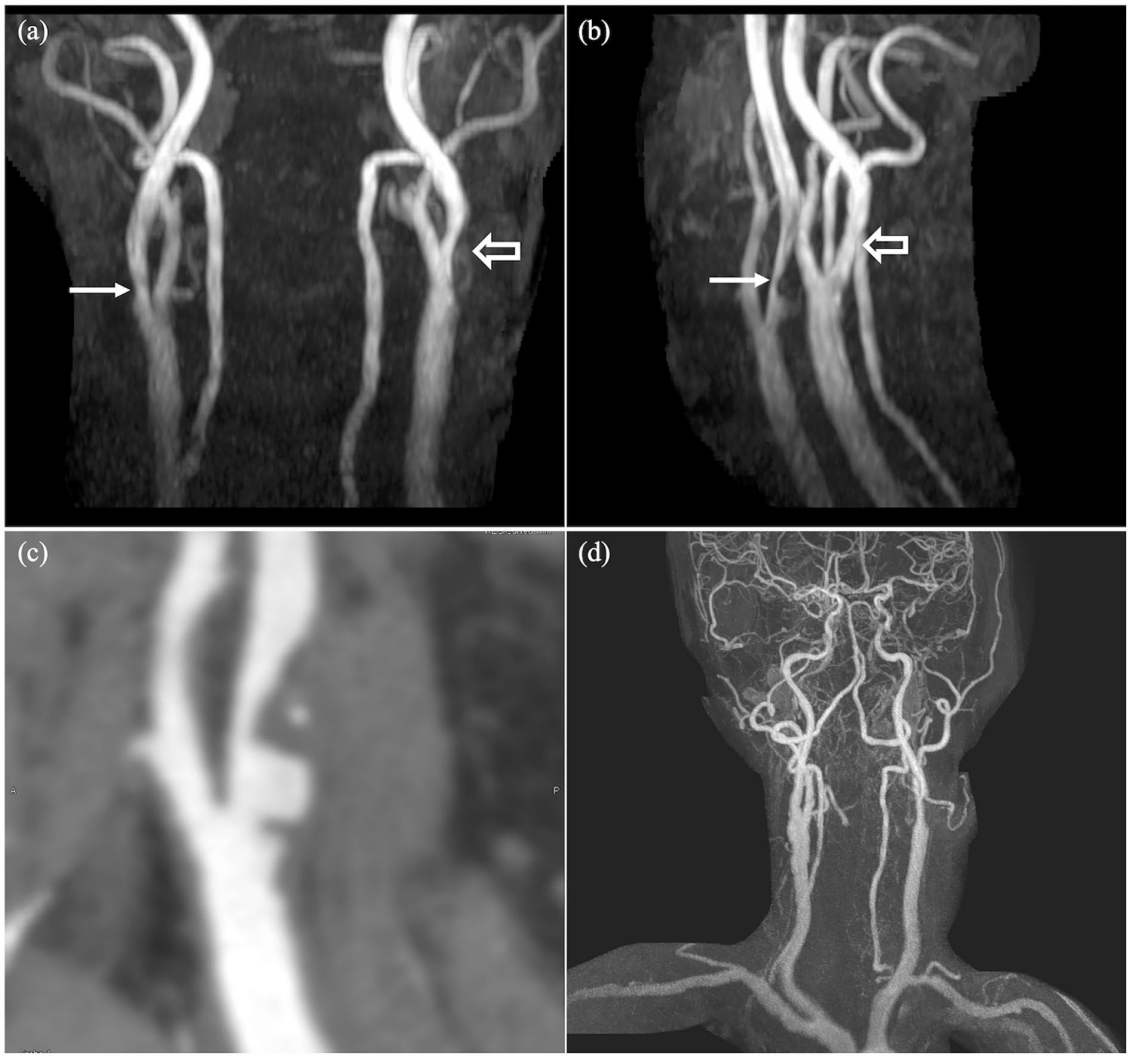
An incidentally detected significant extracranial stenosis in a 71 year-old-male with cognitive impairment. A severe stenosis at right proximal ICA (arrows) and a mild stenosis at left proximal ICA (open arrows) are noted on TOF-MRA **(a,b)**. CT angiography demonstrates non-calcified plaque at right proximal ICA resulting in severe stenosis **(c)**. Contrast-enhanced MR angiography after carotid endarterectomy shows successful dilatation of the right ICA **(d)**.

A total of 92 patients (31.3%, 92/294) received either medical, interventional, or surgical treatment after TOF-MRA. A total of eighty-one patients were prescribed with either antiplatelet medications (*n* = 68, aspirin or clopidogrel) or lipid lowering agent (*n* = 71, statin) once significant stenoses were detected on TOF-MRA. Of note, 26 patients underwent maximal medical therapy for atherosclerosis (aspirin + clopidogrel + statin). In total, fifteen patients (aneurysm: 11 patients, stenosis: 4 patients) were further treated with endovascular intervention (coiling) or surgery (surgical clipping or carotid endarterectomy) (Figures 2, 3). The characteristics of the treated aneurysms and stenoses are provided in the Supplementary Tables 3, 4. Thus, the number needed to scan was 19 for identifying one patient requiring treatment for vascular lesion.

### Risk factors for positive TOF-MRA findings

Table 4 shows the risk factor analysis of 10 potential covariates for positive TOF-MRA findings (aneurysm or stenosis). Being female was an independent risk factor for intracranial aneurysm [odds ratio (OR) 2.05, 95% CI 1.33–3.16; *p* = 0.001]. Conversely, being male was an independent risk factor for significant extra- or intracranial artery stenosis (OR 1.52, 95% CI 1.03–2.24; *p* = 0.03). Old age was associated with both aneurysm (OR 1.04, 95% CI 1.02–1.06; *p* = 0.001) and significant stenosis (OR 1.06, 95% CI 1.04–1.09; *p* < 0.001). Among the vascular risk factors, hypertension (OR 1.78, 95% CI 1.12–2.82; *p* = 0.01) and ischemic heart disease history (OR 2.65, 95% CI 1.59–4.42; *p* < 0.001) were associated with significant artery stenosis. Hypertension showed association with intracranial aneurysm and diabetes with significant stenosis in in univariate analysis but not in multivariate analysis after adjusting for other variables (hypertension: OR 1.19, 95% CI 0.78–1.83; *p* = 0.42; diabetes: OR 1.30, 95% CI 0.87–1.94; *p* = 0.21).

**Table 4:** Risk factors for positive findings on TOF-MRA in patients with cognitive impairment.

**Parameters**	**Intracranial aneurysm (114/1,315 patients)**						**Significant stenosis (121/1,315 patients)**					
	**Univariate analysis**			**Multivariate analysis**			**Univariate analysis**			**Multivariate analysis**		
	**OR**	**95% CI**	***P*-value**	**OR**	**95% CI**	***P*-value**	**OR**	**95% CI**	***P*-value**	**OR**	**95% CI**	***P*-value**
**Demographics**												
Age	1.04	1.02–1.07	<0.001	1.04	1.02–1.06	0.001	1.07	1.05–1.10	<0.001	1.06	1.04–1.09	<0.001
Sex*	2.07	1.35–3.20	0.001	2.05	1.33–3.16	0.001	1.46	1.00–2.12	0.05	1.52	1.03–2.24	0.03
**Vascular risk factors**												
Hypertension	1.45	0.96–2.18	0.08	1.19	0.78–1.83	0.42	2.53	1.63–3.95	<0.001	1.78	1.12–2.82	0.01
Dyslipidemia	0.89	0.59–1.34	0.58				0.95	0.64–1.41	0.80			
Diabetes mellitus	1.03	0.68–1.56	0.89				1.64	1.12–2.39	0.01	1.30	0.87–1.94	0.21
Smoking	0.78	0.50–1.24	0.29				1.21	0.81–1.82	0.35			
Alcohol	0.74	0.48–1.12	0.15				1.28	0.87–1.87	0.21			
Obesity	0.71	0.22–2.33	0.57				1.77	0.78–4.05	0.18			
Previous stroke	0.99	0.47–2.10	0.98				1.51	0.80–2.86	0.20			
Previous ischemic heart	1.37	0.73–2.58	0.33				3.53	2.16–5.79	<0.001	2.65	1.59–4.42	<0.001
disease												

* For analysis, male sex was set as a baseline for intracranial aneurysm while female sex for significant stenosis.

**Figure 4:**
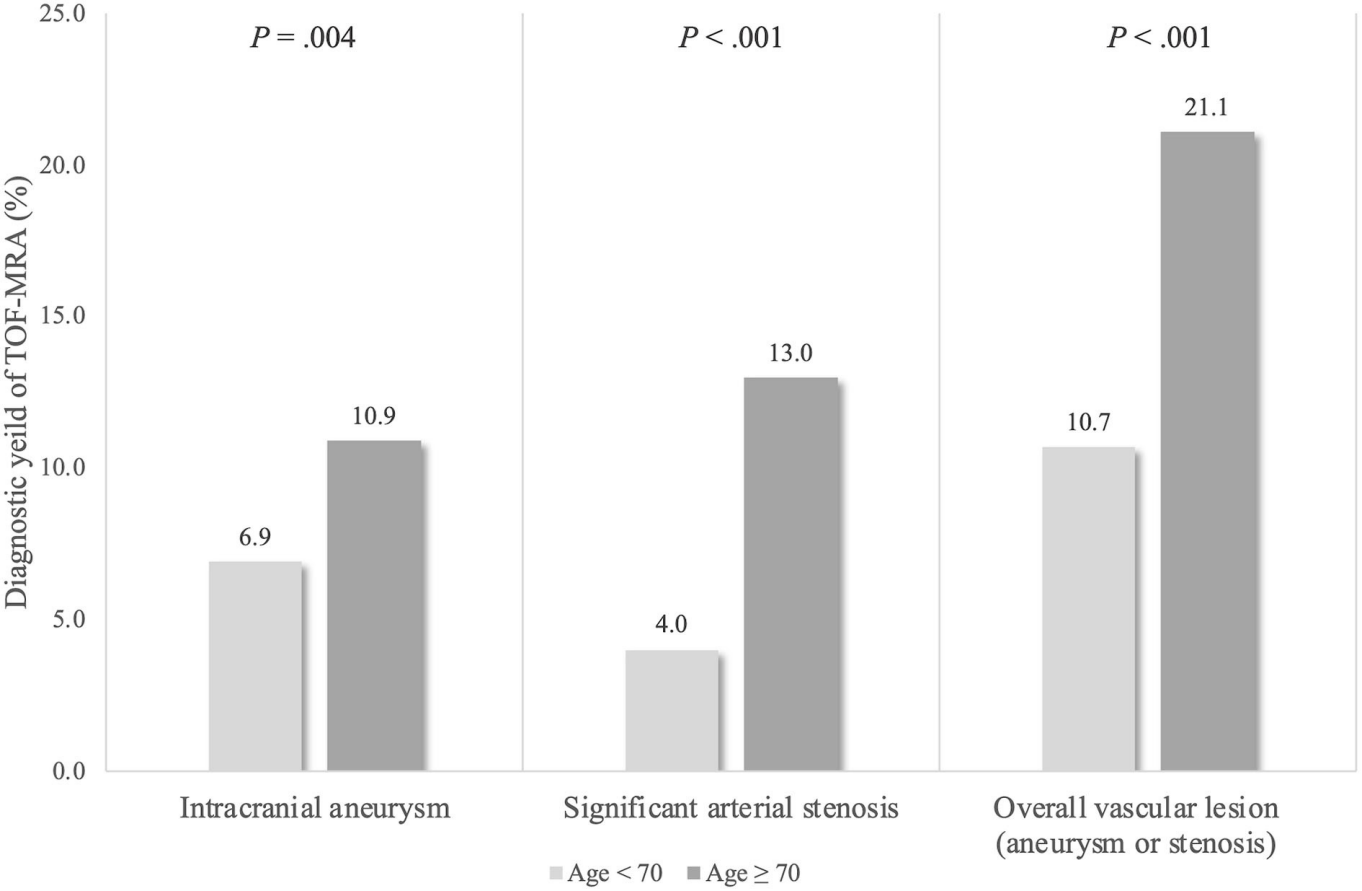
Diagnostic yield of TOF-MRA for detection of intracranial aneurysm or significant extra- and intracranial artery stenosis based on age stratification. The diagnostic yield of TOF-MRA was significantly higher in patients with age ≥ 70.

When the age cutoff was set at 70 (mean age of the study population), the diagnostic yield of TOF-MRA for incidental vascular lesions was significantly higher in older patients (age ≥ 70) [21.1% (216/1,026) vs. 10.7% (78/727), *p* < 0.001)] (Figure 4). Likewise, the diagnostic yield of TOF-MRA for significant arterial stenoses was significantly higher in patients having hypertension or ischemic heart disease history than patients without those risk factors [12.0% (97/810) vs. 4.8% (24/505), *p* < 0.001].

## Discussion

Time-of-flight magnetic resonance angiography is currently an optional sequence for the work-up of patients with cognitive impairment (6, 7). Our results demonstrated an overall 16.8% diagnostic yield of this sequence for detecting incidental vascular lesions in patients with cognitive impairment. The patients with vascular lesions showed worse cognitive function (MMSE 24.2 vs. 22.5, *p* < 0.001). Nearly a third of the patients (92/294) required an additional treatment due to the positive findings on TOF-MRA.

The number of patients visiting dementia clinics is increasing due to increased life expectancy (25). However, incidental vascular findings in these patients are not well documented in the literature. Our study demonstrated a high diagnostic yield of TOF-MRA (9.2% for both aneurysms and significant stenoses) among the patients with cognitive impairment. Although we did not obtain the diagnostic yield of TOF-MRA in elderly control, the above figures are noteworthy considering that the prevalence of intracranial aneurysm or significant stenosis is much lower in the previous studies of general elderly population [prevalence of unruptured intracranial aneurysm: 3.0% in age ≥ 80; asymptomatic intracranial stenosis: 5.9% (mean age 62); asymptomatic carotid stenosis: 5.7% and 4.4% in men and women age ≥ 80, respectively] (26–28). Among the 294 patients with incidental vascular lesions, 92 patients underwent further management. A total of eighty-one patients started medical therapy after the detection of vascular stenosis on TOF-MRA and were prescribed with aspirin, clopidogrel, or statin. In addition, surgery or endovascular intervention was performed on 15 patients. Thus, the number needed to scan for identifying one patient for the treatment of vascular lesion was 19. Our findings indicate the potential benefit of the inclusion of TOF-MRA, because it changed the management plans in considerable number of patients (31.3%, 92/294). However, there were still large number of patients (68.7%, 202/294) who did not receive any of the treatment. Among them, 59 patients were followed up with CT or MR angiography although no further treatment was performed due to a stable disease course or poor general condition. We suggest that future study should focus on cost-effectiveness analysis to justify the routine use of TOF-MRA in patients with cognitive impairment.

Evidence regarding the association between cognitive impairment and intracranial aneurysm is lacking. Few studies focusing on unruptured intracranial aneurysms reported that a non-negligible proportion of patients had cognitive impairment even before undergoing treatment for aneurysm (12, 13). Association between intracranial aneurysm and atherosclerosis and between atherosclerosis and dementia is well established in the previous studies (29–31). Therefore, the high prevalence of intracranial aneurysm in cognitive impairment patients may be explained by a common pathologic process (i.e., atherosclerosis). Several studies suggest the implication of smoking and hypertension since they are well-known risk factors for the development of intracranial aneurysm and vascular cognitive impairment (32). In our study, risk factor analysis showed that being female and old age are the two independent risk factors, supporting the previous findings (14–16). However, we obtained contradictory results regarding hypertension and smoking as risk factors for intracranial aneurysm. Although hypertension showed the significant association with intracranial aneurysm in univariable analysis, this did not apply in multivariable analysis. These findings imply a more complex underlying mechanism of association between cognitive impairment and intracranial aneurysm, rather than mere sharing of the common risk factors. Further studies are warranted to evaluate the association between the two disorders and the underlying mechanism thereof.

Compared to healthy subjects, patients with cognitive impairment are more likely to exhibit significant extra- or intracranial artery stenosis (8–10). It is well known that atherosclerosis increases the risk of vascular dementia (33). Not only vascular dementia but also Alzheimer's disease is associated with atherosclerosis as reported in a study demonstrating the correlation between atherosclerotic burden and neuritic plaque on brain pathology (11). Moreover, faster Alzheimer's disease progression has been reported in patients with accompanying atherosclerotic disease (34). However, the pathophysiology of atherosclerosis-induced cognitive impairment has not yet fully answered (35). Cerebral changes caused by silent embolization, inflammation, or hypoperfusion were suggested as the potential mechanism in the previous studies, albeit the population-based study designs have limitation in establishing the exact causal relationship (36, 37). Risk factor analysis in our study demonstrated that being male, old age, hypertension, and ischemic heart disease history were the independent risk factors for significant stenosis, which is in concordance with previous findings (17–19). Notably, the highest odds ratio was observed for the risk factor of ischemic heart disease history. This may be attributable to the fact that coronary artery disease and cerebral artery stenosis share common risk factors (38).

Our study has several limitations. First, we did not take into account whether the incidentally detected aneurysms or significant arterial stenoses altered the course of cognitive impairment. This would be an important question that needs to be answered in the future studies to validate the clinical implication of TOF-MRA in patients with cognitive impairment. Second, as this was a retrospective study, risk factor data were not available for 438 patients, leading to their exclusion from the risk factor analysis. Notably, the excluded patients were younger (67.4 vs. 71.2 years; *p* < 0.001). This might have caused bias in our study results. However, no significant difference was observed between the two groups regarding sex, intracranial aneurysm, and significant stenosis proportion. Third, there may be a potential risk of selection bias in our study because whether to perform TOF-MRA or not was determined by the physicians' preference considering the patients' vascular risk factors such as hypertension, diabetes mellitus, or coronary artery disease. Indeed, 82.3% (1,442/1,753) of the included patients had at least one vascular risk factors. This may have resulted in overestimation of the diagnostic yield of TOF-MRA for detection of significant arterial stenosis. Additionally, high proportion of the included patients had hypertension (58.7%) and history of smoking (27.1%), which may have resulted in high incidence of intracranial aneurysm as well. However, reviewing the patients with cognitive impairment who did not undergo TOF-MRA, we found the similar proportion of the patients with at least one vascular risk factors (81.6%, 4,811/5,987). Although the proportion of the patients with hypertension, diabetes, dyslipidemia, or obesity was significantly higher in the group that underwent TOF-MRA, the absolute difference was not that large to fully explain the high diagnostic yield of TOF-MRA in this group (Supplementary Table 2). It is known that if the sample size is large enough, even small difference in any effect can produce a small *p*-value (39, 40). Therefore, the statistical significance despite the relatively small absolute difference may be resulted from the large sample size in our study. In addition, the diagnostic yield for the detection of intracranial aneurysm (9.2%) was much higher than the previous study from our institution (2.8% in normal elderly population with age ≥ 70) (41). This large difference may be due to not only the presence of cognitive impairment, but also the difference in MR magnet strength or size of aneurysm between the two studies. A 3.0T MR machine was used in our study, which is known to better depict small aneurysms (<3 mm) than a 1.5T MR machine (42, 43).

Fourth, our study population was from the single tertiary center. The diagnostic yield of TOF-MRA may differ between institutions because the characteristics of patient groups may vary by medical centers. Nevertheless, our study has a strength in that we recruited a large cohort for analysis. Fifth, half of the intracranial aneurysms in our study were below 3 mm. When evaluating an aneurysm of this size, difficulty in distinguishing it from a vascular infundibulum may arise. This is an important issue because inclusion of vascular infundibula could overestimate our results. To prevent the issue, two reviewers referred to the source images of TOF-MRA (1-mm thickness) when there was any ambiguity in distinguishing between the two entities on projection images. Finally, other less common vascular lesions were not included in the study. On retrospectively reviewing the radiologic reports, we found three patients with suspected dural arteriovenous fistula and one patient with a small arteriovenous malformation. However, none of the patients underwent transfemoral cerebral angiography for confirmatory diagnosis.

In conclusion, our study demonstrated the potential benefit of 3D TOF-MRA, given that it showed high diagnostic yield for detecting vascular lesions in patients with cognitive impairment and the considerable number of these lesions required further treatment. A 3D TOF-MRA may be included in the routine MR protocol for the work-up of this patient population, especially in older patients and patients with vascular risk factors.

## Data availability statement

The original contributions presented in the study are included in the article/Supplementary material, further inquiries can be directed to the corresponding author.

## Ethics statement

The studies involving human participants were reviewed and approved by Institutional Review Board of Asan Medical Center. Written informed consent for participation was not required for this study in accordance with the national legislation and the institutional requirements.

## Author contributions

HP: curated and analyzed data and drafted manuscript. CS: design and conceptualized study and revised manuscript. WS, HH, and WK: collected and constructed database. J-SL, J-HL, HK, and SK: provided conceptual feedback and revised manuscript. All authors contributed to the article and approved the submitted version.

## Funding

This work was supported by the National Research Foundation of Korea and Korea Health Industry Development Institute (NRF-2021R1C1C1014413 and HI18C2383).

## Conflict of interest

The authors declare that the research was conducted in the absence of any commercial or financial relationships that could be construed as a potential conflict of interest.

## Publisher's note

All claims expressed in this article are solely those of the authors and do not necessarily represent those of their affiliated organizations, or those of the publisher, the editors and the reviewers. Any product that may be evaluated in this article, or claim that may be made by its manufacturer, is not guaranteed or endorsed by the publisher.
