# Supplementary material for: Diagnostic yield of TOF-MRA for detecting incidental vascular lesions in patients with cognitive impairment: An observational cohort study
Source: Front Neurol. 2022 Aug 25;13:958037. doi: 10.3389/fneur.2022.958037 (PMC9453548; doi:10.3389/fneur.2022.958037)
Supplement: Supplementary file 1 [file Data_Sheet_1.docx]

**Supplemental Materials**

***MRI Analysis***

All evaluation of intracranial aneurysms was performed on maximal intensity projection images (MIP). However, in case of tiny aneurysm (< 3 mm), source images were referred. The locations of aneurysms were categorized as follows: internal carotid artery (ICA); anterior, middle, and posterior cerebral arteries (ACA, MCA, and PCA, respectively); anterior and posterior communicating arteries; and vertebral and basilar arteries (VA and BA, respectively). The degree and location of extra- and intracranial artery stenoses were documented. For measuring carotid bulb stenosis, NASCET criteria was used (1). For measuring intracranial artery stenosis, the percentage decrease in the diameter of most stenotic portion compared to that of proximal normal artery was calculated (2).

**References**

1. Ferguson GG, Eliasziw M, Barr HW, Clagett GP, Barnes RW, Wallace MC, et al. The North American Symptomatic Carotid Endarterectomy Trial: Surgical Results in 1415 Patients. *Stroke* (1999) 30(9):1751-8.

2. Samuels OB, Joseph GJ, Lynn MJ, Smith HA, Chimowitz MI. A Standardized Method for Measuring Intracranial Arterial Stenosis. *American journal of neuroradiology* (2000) 21(4):643-6.

**Supplemental Tables**

**eTable 1. Sequence parameters for MR protocols**

| Parameter | 3D T1WI | T2WI | T2 FLAIR | SWI | DTI | TOF-MRA (Intracranial vessels) | TOF-MRA (Neck vessels) |
| --- | --- | --- | --- | --- | --- | --- | --- |
| Sequences | 3D, FFE | 2D, TSE | 2D, TSE | 3D, FFE | EPI | 3D, FFE | 3D, FFE |
| TE (ms) | 2.9 | 3000 | 125 | 7.2 | 78 | 3.5 | 3.5 |
| TR (ms) | 6.5 | 80 | 9000 (TI^*^ 2500) | 31 | 4013 | 21 | 25 |
| Flip angle (°) | 9 | 90 | 90 | 17 | 90 | 20 | 20 |
| FOV (mm)  (RL × AP × FH)^†^ | 211 × 256 × 256 | 220 × 220 | 220 × 220 | 179 × 220 × 146 | 224 × 224 × 140 | 200 × 200 × 108 | 150 × 150 × 100 |
| Voxel (pixel) size (mm) | 1.0 × 1.0 × 1.0 | 0.4 × 0.4 | 0.9 × 0.9 | 0.4 × 0.4 × 2.0 | 1.8 × 1.8 × 2.0 | 0.4 × 0.4 × 0.6 | 0.3 × 0.3 × 1.0 |
| Number of slices | 211 | 38 | 38 | 73 | 70 | 180 | 100 |
| Slice orientation | Sagittal | Axial | Axial | Axial | Axial | Axial | Axial |
| Slice thickness (mm) | 1 | 4 | 4 | 2 | 2 | 0.6 | 1 |
| Note.—* Inversion time. † Right/left; anterior/posterior; and foot/head. TOF-MRA = time-of-flight magnetic resonance angiography. T1WI = T1-weighted image. T2WI = T2-weighted image. FLAIR = fluid-attenuated inversion recovery image. SWI = susceptibility-weighted image. DTI = diffusion tensor image. FFE = fast field echo. TSE = turbo spin echo. EPI = echo-planar imaging. TE = echo time. TR = repetition time. FOV = field of view | | | | | | | |

**eTable 2. Characteristics of patients between the groups that underwent and did not undergo TOF-MRA.**

| Characteristics | Patients with cognitive impairment who underwent TOF-MRA (n = 1753) | Patients with cognitive impairment who did not undergo TOF-MRA (n = 5897) | P value |
| --- | --- | --- | --- |
| Sex (n) |  |  | .002 |
| Female | 1044 (59.6%, 1044/1753) | 3778 (64.1%, 3778/5897) |  |
| Male | 709 (40.4%, 704/1753) | 2119 (35.9%, 2119/5897) |  |
| Age (years) | 70.2 ± 10.6 | 69.9 ± 10.9 | .36 |
| Education (years) | 9.7 ± 5.4 | 9.9 ± 5.3 | .21 |
| MMSE (0–30 points) | 23.9 ± 5.5 | 23.5 ± 5.4 | .02 |
| GDS (1–7 scales) | 3.2 ± 2.1 | 3.2 ± 1.1 | .42 |
| CDR (0–3 scales) | 0.6 ± 0.5 | 0.6 ± 0.5 | .35 |
| Vascular risk |  |  |  |
| Hypertension | 980 (58.7%, 980/1669) | 2748 (50.4%, 2748/5454) | < .001 |
| Diabetes | 513 (31.1%, 513/1651) | 1338 (24.8%, 1338/5393) | < .001 |
| Dyslipidemia | 567 (38.1%, 567/1490) | 1659 (32.5%, 1659/5101) | < .001 |
| Smoking | 419 (27.1%, 419/1548) | 1236 (24.5%, 1236/5046) | .08 |
| Alcohol | 555 (36.3%, 555/1529) | 1861 (37.0%, 1861/5025) | .60 |
| Obesity | 59 (3.9%, 59/1501) | 117 (2.3%, 117/5021) | .002 |
| Previous stroke | 110 (7.6%, 110/1453) | 417 (8.4%, 417/4961) | .36 |
| Ischemic heart disease | 127 (8.8%, 127/1448) | 380 (7.8%, 380/4852) | .36 |
| Note.—Values denote mean ± standard deviation and number (%). Due to missing data, the denominators for each vascular risk factor were different. MMSE = Mini-Mental State Examination. GDS = Global Deterioration Scale. CDR = Clinical Dementia Rating | | | |

**eTable 3. Characteristics of the treated aneurysms**

| Patients | Number of aneurysms | Size (mm) | Location | Bleb | Treatment |
| --- | --- | --- | --- | --- | --- |
| #1 | 1 | 4.5 | MCA | Absent | Coiling |
| #2 | 1 | 3 | ICA | Absent | Coiling |
| #3 | 1 | 6.5 | MCA | Absent | Coiling |
| #4 | 1 | 5 | MCA | Absent | Coiling |
| #5 | 3 | 6, <3, <3 | Pcom, ICA | Absent | Coiling |
| #6 | 2 | 12, 8 | Pcom | Present | Clipping and coiling |
| #7 | 1 | 4 | MCA | Absent | Coiling |
| #8 | 1 | 4.5 | ACA | Absent | Coiling |
| #9 | 1 | 8 | ICA | Absent | Coiling |
| #10 | 1 | 3.5 | ACA | Absent | Coiling |
| #11 | 2 | 8, 3 | BA, Acom | Absent | Coiling |
| MCA = middle cerebral artery. ICA = internal carotid artery. Pcom = posterior communicating artery. ACA = anterior cerebral artery. BA = basilar artery. Acom = anterior communicating artery. | | | | | |

**eTable 4.** Characteristics of the treated stenoses

| Patients | Location | Grade | Treatment |
| --- | --- | --- | --- |
| #1 | Carotid bulb | Severe stenosis | Endarterectomy |
| #2 | Carotid bulb | Moderate stenosis | Endarterectomy |
| #3 | Carotid bulb | Severe stenosis | Endarterectomy |
| #4 | Carotid bulb | Occlusion | Endarterectomy |
|  | | | |
